# Dietary Black Soldier Fly Larvae Meal and Its Impact on the Growth Performance and Gut Health of Broilers Under an Intestinal Challenge

**DOI:** 10.3390/metabo15060347

**Published:** 2025-05-23

**Authors:** Yuri Katagiri Dalmoro, Guilherme Librelotto de Godoy, Jessica Cristina Agilar, Glauco Anderson Raddatz, Fernanda de Candido de Oliveira, Natieli Witt, Catarina Stefanello

**Affiliations:** 1 Department of Animal Science, Federal University of Santa Maria, Santa Maria 97105-900, RS, Brazil; yuri.katagiri@acad.ufsm.br (Y.K.D.); guilherme.godoy@acad.ufsm.br (G.L.d.G.); jessica.agilar@acad.ufsm.br (J.C.A.); glauco.raddatz@acad.ufsm.br (G.A.R.); natielli.witt@acad.ufsm.br (N.W.); 2 Department of Food Science and Technology, Federal University of Santa Maria, Santa Maria 97105-900, RS, Brazil; fernanda.candido@acad.ufsm.br

**Keywords:** broiler chicken, *Hermetia illucens*, interleukin, lauric acid, short-chain fatty acid

## Abstract

Background/Objectives: The use of black soldier fly (BSF) larvae meal in poultry nutrition is gaining attention as a sustainable protein source with a high nutritional value, an efficient bioconversion of organic waste, and potential functional benefits for intestinal health. This study evaluated the dietary effects of including 5% BSF larvae meal on the growth performance, nutrient digestibility, and energy utilization as well as on the intestinal integrity, gene expression, lipid profile, and short-chain fatty acid (SCFA) production of broilers under an intestinal challenge. Methods: Eight hundred one-day-old male broilers were assigned to four dietary treatments with eight replicates (25 birds/pen) and reared until day 40. Birds were fed either a Basal corn–soy diet or a BSF diet (5% BSF larvae meal replacing energy- and protein-yielding ingredients). Diets were provided to a non-challenged group and a challenged group, which was orally gavaged with *Eimeria* spp. on day 1 and *Clostridium perfringens* on days 11 and 14. The growth performance was evaluated up to day 40, while the nutrient digestibility, meat lipid profile, intestinal histomorphology, and gene expression were assessed at 21 days. The SCFAs were determined at both 21 and 40 days. Results: It was observed that the intestinal challenge induced dysbiosis and negatively affected growth performance, whereas the BSF meal inclusion partially mitigated these adverse effects. Broilers fed the BSF larvae meal showed increased cecal SCFA concentrations and a lower interleukin-6 gene expression, along with higher lauric and myristic acid levels in breast muscle (*p* ≤ 0.05). Conclusions: The inclusion of 5% BSF larvae meal improved performance without impairing nutrient digestibility or intestinal histomorphology, while increasing cecal concentrations of butyric and acetic acids and promoting a beneficial lipid deposition.

## 1. Introduction

Poultry meat holds substantial economic importance worldwide and represents one of the most consumed sources of animal-derived protein, contributing to a complex and highly interconnected market. In poultry production systems, corn and soybeans are the primary feed ingredients, resulting in extensive land use and significant natural resource consumption. Consequently, increasing attention has been directed toward the development of sustainable strategies for the use of feed ingredients in poultry production systems [1].

Research on *Hermetia illucens*, commonly known as the black soldier fly (BSF), has evolved beyond forensic entomology and pest control, expanding its application as a feed ingredient due to its remarkable bioconversion capacity [2]. The BSF larvae can convert a wide range of organic materials into a nutrient-dense feed ingredient rich in protein and energy while simultaneously reducing land use and carbon dioxide emissions. Additionally, the byproduct of this bioconversion process, known as frass, can be repurposed as fertilizer, further enhancing its economic significance. The nutritional composition of BSF larvae is highly variable and influenced by factors such as the larval stage, rearing substrate, and oil extraction. The protein content in BSF larvae meal can reach up to 60%, while the lipid content may comprise up to 30%, and dry matter levels range between 20% and 44% [3,4]. The combination of a high bioconversion efficiency and nutritional value highlights BSF larvae as a promising ingredient for sustainable livestock production. Previous studies have demonstrated their potential for the partial replacement of soybean meal (SBM), with inclusion levels up to 15% reported to enhance broiler performance. Additionally, this ingredient has been shown to effectively replace fishmeal [5,6] and oils [7,8] in poultry diets and has been incorporated into the diets of free-range poultry systems [9,10].

Beyond its nutritional benefits, BSF meal also exhibits antimicrobial properties, which are aligned with current industry trends [11,12]. The ability of BSF larvae to thrive in microbial-rich environments has driven the synthesis of bioactive compounds with functional properties. Among these, chitin is a nitrogenous polysaccharide forming the exoskeleton of arthropods that exerts immunomodulatory and antimicrobial effects [13]. Lauric acid has demonstrated antimicrobial activity, particularly against Gram-positive bacteria, but also against certain Gram-negative species. Additionally, BSF hemolymph contains a wide range of antimicrobial peptides (AMPs), including defensins, cecropins, and attacins, which contribute to the larva’s defense mechanisms [14]. Despite the well-documented nutritional functions of the BSF as a feed ingredient, its effects on gut health in poultry under intestinal challenge conditions remain insufficiently explored. Notably, insect-derived bioactive compounds, such as AMPs, fatty acids, and chitin, may exhibit bactericidal activity by modulating the host immune response, warranting further investigation. Additional research is needed to elucidate the potential benefit of BSF-derived products in enhancing intestinal integrity, gut health, and overall poultry performance.

The starting hypothesis in the present study was that dietary BSF larvae meal would enhance the performance and intestinal health parameters of broiler chickens during dysbiosis. Therefore, the objective of this study was to evaluate the effects of including 5% BSF larvae meal in corn–SBM diets for broiler chickens up to day 40. The effects were assessed on the growth performance, nutrient digestibility, energy utilization, intestinal integrity, gene expression, short-chain fatty acid production, and lipid profile of broilers under an intestinal challenge with *Eimeria* spp. and *Clostridium perfringens*.

## 2. Materials and Methods

All procedures involving live birds were approved by the Ethics and Research Committee of the Federal University of Santa Maria, Santa Maria, RS, Brazil.

### 2.1. Broiler Husbandry

A total of 800 one-day-old male chicks, Cobb 500, were allocated into 32 experimental floor pens (11 birds/m^2^) in a conventional poultry house. Each pen was equipped with a pendular drinker and one tube feeder, providing broilers ad libitum access to water and mash feed. New wood shavings served as litter. The environmental temperature was maintained according to the guidelines provided by the genetic supplier [15] to ensure thermal comfort throughout the experimental period. Thermal control was achieved with heaters and fans when needed. A continuous lighting program was applied until day 14, followed by a 16L:8D program.

Chicks were purchased from a commercial hatchery, vaccinated for Marek’s disease at the hatchery, and, then, weighed and distributed by body weight (BW) in each pen, using a completely randomized design from 1 to 40 days of age. Chicks were individually weighed in groups of 25 birds per pen at placement, ensuring that the variation in live weight within each experimental unit did not exceed 3%. Birds were then allocated to four treatments, with eight replicate pens of 25 birds each, distributed in a factorial 2 × 2 arrangement composed by 2 diets (Basal or BSF), non-challenged, or experimental challenged groups.

### 2.2. Dietary Treatments

Broilers were subjected to a three-phase feeding program comprising starter (1 to 14 days), grower (14 to 28 days), and finisher (28 to 40 days) diets formulated based on Brazilian poultry and swine tables [16]. The diets consisted primarily of corn and SBM sourced from the same batch and analyzed prior to feed formulation. Broilers were divided into two groups receiving different diets: Basal diet (Basal), formulated with corn and SBM, or BSF diet (BSF), including 5% BSF larvae meal, corn, and SBM. The BSF larvae meal was obtained from Nutrition Technologies Sdn Bhd (Johor, Malaysia). Its composition was analyzed using AMINONIR^®^ (Evonik Operations GmbH, Hanau-Wolfgang, Germany), while digestibility values were based on company recommendations and adjusted according to a previous publication [17]. The analyzed gross energy (GE) and chemical composition of BSF larvae meal is presented in Table 1.

The BSF larvae meal replaced energy- and protein-yielding ingredients according to its nutritional matrix. Ingredient and nutrient composition of Basal or BSF experimental diets is presented in Table 2.

Experimental feeds were prepared in a horizontal mixer with 400 kg capacity (UFSM, Santa Maria, RS, Brazil), and the equipment was vacuumed between batches. Each experimental diet was provided to groups of broilers that were non-challenged or subjected to an *Eimeria* spp. and *C. perfringens* challenge to complete the four treatments.

On day 1, all birds in the challenged group were individually gavaged with 10× the recommended dose of a commercial coccidiosis vaccine containing *Eimeria acervulina*, *E. brunetti*, *E. maxima*, *E. mitis*, *E. necatrix*, *E. praecox*, and *E. tenella*. At 11 and 14 days of age, chicks in the challenged group were also individually orally gavaged with *C. perfringens* (1 × 10⁸ CFU/mL; 1 mL/bird per day; MercoLab, Cascavel, PR, Brazil). The challenge was selected based on previous research [18,19].

### 2.3. Fatty Acids of Experimental Diets

Table 3 presents the fatty acid profile in each experimental diet, expressed as a percentage of the total extract. The method utilized to determine the fatty acids was a one-step methylation approach. In summary, 0.5 g of each sample was subjected for simultaneous extraction and transesterification by incubating with toluene and 5% methanolic HCl for 2 h at 70 °C. Nonadecanoic acid (C19:0; Sigma-Aldrich, Munich, Germany) was added as an internal standard. Following methylation, 5 mL of 6% potassium carbonate solution and 2 mL of toluene were added. After centrifugation, 1 mL of the upper organic layer was transferred into a gas chromatograph (GC) vial for analysis. The GC analysis was performed using an Agilent 7890A system (Agilent Technologies, Santa Clara, CA, US), equipped with an Agilent 7693 autosampler and G4514A injection module. Separation of methyl esters was carried out on an Agilent HP-88 capillary column (100 m × 0.25 mm internal diameter, 0.20 µm film thickness), with hydrogen as the carrier gas at a constant pressure of 11 psi. The flame ionization detector was operated with flow rates of 35 mL/min for hydrogen and 350 mL/min for synthetic air, at 260 °C. Injection parameters included a temperature of 250 °C, a split ratio of 50:1, and a 1 µL injection volume. The oven program started at 100 °C (5 min hold), followed by an increase of 4 °C/min to 240 °C, which was maintained for 30 min, totaling a 70 min runtime. Fatty acid methyl esters (FAMEs) were identified by comparing retention times with those of a commercial standard mix (Supelco 37 Component FAME Mix). Chromatographic data were processed using Agilent OpenLab software version 3.6 (Agilent Technologies, Santa Clara, CA, US).

### 2.4. Growth Performance

The BW and feed intake (FI) of the birds were recorded per replicate and measured weekly on days 1, 7, 14, 21, 28, 35, and 40. Feed conversion ratio (FCR; feed/gain), corrected for mortality, and body weight gain (BWG) were calculated on a weekly basis, as well as per feeding phase and for the entire period. The weight of dead birds was recorded daily.

### 2.5. Intestinal Permeability and Ileal Digestibility

At 21 days of age, one bird per pen (with the average weight of the experimental unit) received an oral dose of 2.2 mg/bird of FITC-d (systemic fluorescein isothiocyanate–dextran), dissolved in 1 mL of Milli-Q water. Blood samples were collected 1.5 h after gavage, kept at room temperature, and then centrifuged [20]. To determine the FITC-d concentration in serum (μg/mL), the fluorescence levels of the diluted serum (1:1 in phosphate-buffered saline) were measured at an excitation wavelength of 485 nm and an emission wavelength of 528 nm (Synergy HT, Multi-mode microplate reader, BioTek Instruments, Inc., Winooski, VT, US) [18,21]. The FITC-d is used as marker for intestinal integrity since it is a high-molecular-weight molecule that is only detected in the bloodstream when the intestinal mucosa is damaged, indicating permeability alterations.

On day 21, the contents of the distal two-thirds of the ileum were collected from four chickens per experimental unit (with the average BW of the pen). Ileal digesta was collected in plastic containers by flushing with distilled water and pooled by experimental unit. Samples were frozen immediately after collection. Celite at 1% was supplemented in all feeds three days before sample collection. It was used as an indigestible marker for digestibility calculations. Feed and ileal digesta samples were ground and analyzed to determine dry matter (DM), acid-insoluble ash, gross energy, and crude protein (CP).

Nitrogen was measured using the combustion method (Thermo-Finnigan Flash EA 1112, Waltham, MA, US), while gross energy was determined with a bomb calorimeter (IKA Werke C200, Staufen, Germany). Acid-insoluble ash was extracted from both feed and ileal digesta to determine the indigestible marker. Then, ileal digestible energy (IDE) and the apparent ileal digestibility of DM, energy, and CP were calculated [18,22].

Ileal digestibility and IDE were calculated as Digestibility (%) = [1 − (Mi/Mo) × (Eo/Ei)] × 100 and IDE (kcal/kg) = GEi − [GEo × (Mi/Mo)], where Mi represents the concentration of acid-insoluble ash in the diet in g/kg of DM; Mo is the concentration of acid-insoluble ash in the ileal digesta in g/kg of DM; Ei represents the concentration of DM or N in the diet in mg/kg of DM; and Eo is the concentration of DM or N in the ileal digesta in mg/kg of DM. GEi is gross energy (kcal/kg) in the diet; GEo is the gross energy (kcal/kg) in the ileal digesta in g/kg of DM.

### 2.6. Jejunal Histomorphology and Molecular Analysis of Mucosa (qRT-PCR)

On day 21, a segment of medial jejunum was collected from one bird per pen (the same bird used for intestinal permeability analysis). After collection, it was fixed in 10% formaldehyde for 72 h to prevent tissue autolysis. Subsequently, the samples were processed through the stages of dehydration, clearing, and paraffin embedding, using alcohols of different concentrations, xylene, and paraffin, respectively. Once embedded, the tissue was blocked in paraffin and sectioned into 7 µm thick slices using a Thermo Scientific microtome (Microm HM 325, Fisher Scientific Co LLC, Hampton, NH, US). For microscopic analysis, stained slides were imaged using a Zeiss microscope (AxioScope A1-AX10, Carl Zeiss do Brasil Ltd., Sao Paulo, SP, Brazil) equipped with an Axioscan 305 camera. Morphometric analysis was performed using ImageJ version 1.52 software. Hematoxylin and eosin-stained slides were used for morphometric comparison. From each slide, six samples were photographed and four random measurements were taken from each image for both villi and crypts using ImageJ software.

Mucosa was scraped in the medial jejunum from the same four birds slaughtered for ileal digesta collection on day 21. The mucosa from each bird was homogenized with Trizol and stored at −80°C until molecular analysis. The mRNA expression of mucin-2 (MUC2) and interleukin-6 (IL-6) in the jejunum was measured using quantitative real-time PCR (qRT-PCR). Total RNA was extracted following the Tri Reagent protocol. The mRNA samples were treated with Turbo DNase, and RNA concentration was determined spectrophotometrically using a NanoDrop. Subsequently, qRT-PCR was performed. The samples were preserved in Trizol and sent to an external laboratory for analysis to determine jejunal gene expression of MUC2 and IL-6 (IMUNOVA, Curitiba, PR, Brazil). The methodology followed a previous study conducted by the research group [18].

### 2.7. Short-Chain Fatty Acids of Cecal Contents and Lipid Profile of Breast Samples

At 21 and 40 days of age, the cecal content was collected from four birds per experimental unit (same birds used for ileal digestibility and gene expression analysis) and frozen for subsequent analysis of short-chain fatty acids (SCFAs). For SCFA analysis, 1 mL of distilled water was added per gram of cecal content, which was then homogenized in a vortex for 30 s, followed by centrifugation at 11,300× *g* for 5 min [23]. A total of 200 μL of the supernatant and 200 μL of formic acid was added and vortexed again. After centrifugation, 100 μL of the resulting sample was diluted with 100 μL of an internal standard composed of 3-octanol (6.54 mg/L in methanol). Then, 1 µL of this extract was injected into a GC equipped with a flame ionization detector (FID) containing an auto sampler system in split mode (1:10). Hydrogen was used as carrier gas at a constant pressure of 25 psi. The column used was CP-WAX 52 CB (60 m × 0.25 mm; 0.25 µm thickness film). The temperature was adjusted to 80 °C for 1 min, increasing to 120 °C at 8 °C/min, and increasing at 15 °C/min until 230 °C, remaining for 1 min. Injector and detector temperatures were defined to 250 °C. The parameters including selectivity, linearity, linear range, repeatability, precision, limit of detection, and limit of quantification were used to validate the method for the following compounds: acetic, propionic, butyric, isovaleric, and pentanoic acids. The results were expressed in mmol/kg.

Samples of breast meat were also collected for lipid analysis from the same bird at 21 days of age and sent to the Food Analysis Laboratory at UFSM (CTA, Santa Maria, RS, Brazil). For lipid analysis, a total of 4 g of breast sample was collected from each bird at 21 days and placed in a 50 mL Falcon tube, followed by the addition of 16 mL of methanol, 5 mL of water, and 8 mL of chloroform [24]. This solution was homogenized on a shaker table for 1 h for lipid extraction. After this step, 8 mL of chloroform and 8 mL of sodium sulfate (1.5%) were added, followed by homogenization (2 min) and centrifugation. The chloroform fraction containing the lipids was collected, and the chloroform was evaporated under vacuum at 40 °C. The lipids were then subjected to a transesterification procedure to obtain FAMEs, according to the International Organization for Standardization , with some modifications. A total of 500 μL of potassium hydroxide (2 M) and 2 mL of hexane was added in the solution. Then, samples were heated at 40 °C for 5 min, after which the tubes were cooled, and the content containing the FAMEs was transferred to a 2 mL vial. This sample was used in a GC equipped with a flame ionization detector. The extract was injected into the injector port, operating in split mode (1:20) at 250 °C, using hydrogen as the carrier gas at a constant pressure of 22 psi. The FAMEs were separated on a SP-2560 column (100 m × 0.25; 0.20 µm thickness film). The column temperature programming started at 80 °C for 2 min, increasing 8 °C/min until reaching a temperature of 200 °C, then rising at a rate of 1 °C/min to 210 °C, where there was an increase 2 °C/min, until reaching a temperature of 240 °C, remaining in this condition for 3 min. The FID detector was held constant at 250 °C. The identification of the FAMEs was obtained by comparing the retention times of the compounds with a Mix FAME 37 fatty acid standard . Results of each fatty acid and total fatty acids were expressed as a percentage of the total area of the chromatograms, considering the correction factors of the FID and the conversion of esters into acids [25].

### 2.8. Statistical Analysis

A two-way analysis of variance was performed using the MIXED procedure of SAS (version 9.4, SAS Institute Inc., Cary, NC, US), with diet and challenge as the main effects. Mean comparisons were conducted using Fisher’s LSD test. Significant differences among treatment means were declared when *p* ≤ 0.05, while tendencies were declared when 0.05 < *p* ≤ 0.10.

## 3. Results

### 3.1. Growth Performance

The analyzed composition of experimental feeds was according to the expected values. The growth performances of non-challenged or challenged broiler chickens fed Basal or BSF diets are shown in Table 4. No interactions between the diet and challenge were observed in the growth performance throughout the experimental period (*p* > 0.05). Treatments also did not affect the broiler mortality, which averaged 3.0% from 1 to 40 days of age. Broilers on the challenge group had a lower BWG compared to the non-challenged birds from 1 to 7, 7 to 14, 14 to 21, and 21 to 28 days of age (*p* ≤ 0.05). The FI decreased in challenged broilers from 1 to 7 and 7 to 14 days of age (*p* < 0.001). Regarding the FCR, the challenge model showed a poor FCR from 1 to 7, 7 to 14, 14 to 21, and 28 to 35 days of age (*p* ≤ 0.05). No effects of the challenge treatments were observed on the BWG, FI, and FCR from 35 to 40 days of age.

From 1 to 21 and 21 to 40 days, the BWG also reduced in challenged broilers (*p* ≤ 0.05). The FI decreased in challenged birds from 1 to 21 days; however, due to the compensatory response, it increased from 21 to 40 days of age. The highest and the worst FCR was observed in non-challenged broilers from 1 to 21 and 21 to 40 days (*p* ≤ 0.05). In the overall period, the BWG was the lowest in the challenged group (*p* = 0.005), with an increased FI (*p* = 0.039) and the poorest FCR (*p* = 0.009).

Diets formulated with 5% BSF larvae meal did not affect the weekly BWG. Nevertheless, BSF diets tended to decrease the FI from 1 to 7 days (*p* = 0.062) and to improve the FCR from 14 to 21 days (*p* = 0.072). Broilers fed the BSF diet had an improved FCR from 1 to 21 and 21 to 40 days compared to broilers fed the Basal diet. In the overall period, broilers fed the BSF diet also had an improved FCR and higher BWG (*p* ≤ 0.05).

### 3.2. Ileal Digestibility, Intestinal Permeability, Jejunal Histomorphology, and Molecular Analysis of Mucosa (qRT-PCR)

Table 5 demonstrates the effect of the diets and challenge on the apparent ileal digestibility, intestinal integrity, jejunal histomorphology, and gene expression of broiler chickens at 21 days of age. No interactions between the diet and challenge were observed for the intestinal health parameters and ileal digestibility (*p* > 0.05). The intestinal permeability increased when broilers were challenged with *Eimeria* spp. and *C. perfringens* (*p* = 0.041). The jejunal gene expression of IL-6 increased in challenged broilers (*p* = 0.002), while the expression of MUC2 was not affected. Additionally, the challenge model did not affect either the jejunal histomorphology or digestibility of nutrients and energy.

Regarding the BSF inclusion, the ingredient did not influence the intestinal integrity, MUC2, crypt depth, muscular height, and ileal digestibility of broilers at 21 days of age. However, broilers fed the BSF diet had a lower IL-6 jejunal expression and tended to present a higher jejunal villus height and villus–crypt ratio (*p* ≤ 0.05).

### 3.3. Short-Chain Fatty Acids of Cecal Contents and Lipid Profile of Breast Samples

The content of cecal SCFAs is shown in Table 6 and no interactions between the diet and challenge were observed (*p* > 0.05). The challenge model did not affect SCFAs with the exception of the propanoic acid, where an increased content of propanoic acid was observed in challenged broilers at 21 and 40 days of age (*p* ≤ 0.05). Regarding the experimental diets, acetic and butyric acids increased at 21 days when broilers were fed the BSF diet, while only butyric acid increased at 40 days of age (*p* ≤ 0.05).

The lipid profile of breast samples is shown in Table 7. No interactions between the diet and challenge were observed on the lipid profile of breast samples at 21 days (*p* > 0.05). There was no significant difference in the total fatty acids and lipid profile between non-challenged and challenged broilers. The inclusion of the BSF larvae meal in the diets resulted in an increased concentration of lauric and myristic acids in breast samples (*p* = 0.041). However, all other fatty acids were not affected by the dietary treatments.

## 4. Discussion

Regarding the intestinal challenge, the most significant effects of *Eimeria* spp. and *C. perfringens* were a reduced performance, impaired intestinal integrity, and increased IL-6 levels. The growth performance impacts were particularly evident due to dysbiosis, as our objective was to induce dysbiosis without causing severe intestinal lesions or compromising normal bird growth, a goal successfully achieved. Several factors can influence the efficacy of an experimental intestinal challenge model, inducing varying degrees of dysbiosis. These include the types and concentrations of inoculums, when the challenge is observed, the number of gavages, as well as the dietary composition and environmental conditions [26]. In a challenge model where birds were orally gavaged with *C. perfringens* at 5 and 19 days of age, no significant effects were observed on performance [27]. However, using the same inoculum but administering oral gavages at 11, 12, and 13 days of age, greater effects of the proposed challenge were reported on the broiler performance, nutrient digestibility, jejunal histomorphology, and intestinal permeability [18,28]. Depending on the challenge model, birds can compensate for the FI after dysbiosis, as observed in the current study [28]. However, it is possible that intestinal damage may be difficult to repair, leading to a poorer performance also after 21 or 28 days [18].

A previous study indicated that diets with lower levels of BSF meal (3%) exhibited potential prophylactic properties against bacterial dysbiosis and *Salmonella* Gallinarum [29]. An in vitro trial evaluated *C. perfringens’* incubation with digested insect isolates, and it was concluded that BSF-derived protein may be effective against *C. perfringens*. It was evidenced based on the inhibition of bacterial growth, increased SCFA secretion, and alterations in healthy microbiota composition [30]. The present trial and the inclusion level of the BSF larvae meal considered previous results from our systematic review and meta-analysis on the use of *H. illucens* and *T. molitor* in poultry diets [31]. Additionally, this study was motivated by the growing interest in better understanding the functional, prebiotic, and antimicrobial properties of insect larvae compounds, which have been reported to benefit broiler performance and intestinal health [11].

In the present study, diets formulated with 5% BSF meal improved the growth performance, with less pronounced effects on the weekly performance and more expressive impacts on the cumulative performance in broilers challenged with *C. perfringens.* These findings agree with a study evaluating four inclusion levels (0, 4, 8, and 12%) of defatted BSF in broiler diets, where the 4% inclusion was the optimal level for performance [32]. Other studies on defatted BSF larvae meal reported that 10% BSF meal improved the broiler performance [33], whereas a 16% inclusion with the total replacement of SBM tended to reduce the overall performance [34]. Additionally, Schiavone et al. [35] found that a 10% BSF inclusion did not negatively affect bird performance. Similarly, Dabbou et al. [7] observed that the gut morphology and FCR were not adversely impacted by a 15% BSF meal inclusion level.

Most previous studies have replaced SBM with BSF larvae meal, primarily considering it a protein source [7,32,35]. However, certain decisions concerning its dietary inclusion were made prior to the comprehensive characterization of its bromatological composition, digestibility coefficients, and complete nutritional matrix. With detailed data on the ingredient composition and digestible AA, it becomes feasible to apply energy and nutrient matrices to formulate more precise diet formulations. Consequently, the benefits of BSF larvae meal may extend beyond its protein contribution to include functional effects attributed to its content of antimicrobial peptides, fatty acids, and chitin [36].

The BSF-based diet did not affect the nutrient digestibility, jejunal histomorphology, or intestinal permeability. We initially hypothesized that broilers subjected to an intestinal challenge might exhibit improvements in intestinal integrity when fed the BSF diet. However, these differences were not observed, possibly due to dysbiosis, the low BSF inclusion level, or its composition. Despite the growing interest in insect-derived products, data on their antimicrobial and functional properties in mitigating intestinal challenges remain scarce for poultry. Further research is warranted to elucidate the effects of low inclusion levels of insect meal on gut health and the overall performance.

The BSF-based diet formulated in this study contained higher levels of chitin and lauric acid compared to the Basal diet, as presented above. This measurement is particularly relevant, as previous studies indicate that chitin from the insect exoskeleton; the fatty acid profile, especially its high lauric acid content; and AMPs involved in defense mechanisms contribute to the classification of insect meal as a functional ingredient for animal production [13]. The AMPs exert an antimicrobial activity by destabilizing bacterial membranes, disrupting metabolic processes, and/or targeting cytoplasmic components [37]. Additionally, insect-derived fatty acids have been recognized for their antimicrobial functions, which are associated with a cellular pH reduction and dissociation capacity [38].

Yuan et al. [39] fed broilers with 0%, 6%, and 12% BSF larvae meal, with or without a coccidiosis challenge. The authors evaluated cecal interferon-γ (IFN-γ) levels and observed a linear increase in IFN-γ with the inclusion of the BSF meal, although this response varied depending on whether birds were challenged or not. Higher levels of IFN-γ can protect against coccidiosis by inhibiting parasite invasion and survival within host cells. It promotes local inflammation, enhances nitric oxide production, activates cell-mediated cytotoxic responses, and triggers the release of cytoplasmic granules containing perforin and proteases [40]. Immunomodulatory and antimicrobial markers were not assessed in our study; however, de Souza Vilela et al. [41] fed broilers a 20% BSF larvae meal and suggested that the immunomodulatory effects of BSF meal might be attributed to its fatty acid profile, along with the presence of antimicrobial peptides and chitin, which together form an efficient antimicrobial barrier. The authors also reported that this could potentially reduce the requirement for an increased presence of intraepithelial cytotoxic T lymphocytes.

In the current study, the jejunal expression of MUC2 was not affected by either the challenge or diet. Mucin acts as a gut villus barrier, preventing the adhesion of pathogenic microorganisms. Higher BSF inclusion levels in diets (>15%) were previously reported to decrease mucin production, suggesting microbiota changes and mucin dynamics associated with insect meal [42]. On the other hand, IL-6 increased in the challenged group and decreased with the BSF diet. It was previously observed that IL-6 is expressed during *Eimeria* spp. infections and acts as an immune regulator [43]. This effect was shown in a trial with young turkeys fed dietary BSF oil, which reduced the IL-6 expression [44]. The reduction in IL-6 observed in broilers fed BSF-based diets may be attributed to its immunomodulatory potential [11]. Still, the higher lauric acid content detected in both diets and breast samples could be a contributing factor in mitigating dysbiosis, as lauric acid has been shown to inhibit the growth of certain bacteria [45,46].

The microbiota was not analyzed in the present study; however, previous research has investigated the effects of BSF larvae meal on the microbiome, suggesting its influence on digesta fermentation and SCFA production [42]. In general, SCFAs are well known for their numerous beneficial effects on animal gut health and are associated with favorable modifications in the intestinal microbiota composition [1,47,48]. In the present study, increased cecal concentrations of butyric and acetic acids were observed. Similarly, an in vitro study [30] reported an increased SCFA secretion when the fecal-derived microbiota was exposed to digested BSF larvae protein meal or the digestion of chitin-rich BSF larvae protein meal. Butyric acid, as the primary energy source for colonocytes, plays a key role in promoting intestinal epithelial cell differentiation and proliferation. It has also been shown to support gut health, reduce mucosal inflammation, strengthen the intestinal barrier, and regulate gut motility [48].

In the current study, the evaluation of the lipid profile in breast meat samples revealed that only lauric and myristic acids increased with the inclusion of the BSF larvae meal. The fatty acid profile of chicken meat can be influenced by numerous factors, including the dietary composition and the inclusion of lipid sources rich in medium- and long-chain fatty acids, which can enhance their deposition in muscle tissues [49,50]. The BSF larvae meal contains significant amounts of lauric and myristic acids; therefore, it is hypothesized that its inclusion in the diet may modify the fatty acid profile of chicken meat [51]. Lauric acid, when observed in higher concentrations in broiler meat, exhibits antimicrobial activity, particularly against Gram-positive bacteria such as *C. perfringens*, *Staphylococcus aureus*, and *Listeria monocytogenes* [30,52]. This property may help to reduce the bacterial load and to minimize the risk of contamination. Additionally, previous studies reported that meats rich in lauric acid had reduced lipid oxidation, which contributes to product preservation and extends shelf life [53]. In this context, including the BSF meal could indirectly contribute to improving the meat quality by increasing the levels of lauric and myristic acids, even though direct assessments of lipid oxidation were not performed in this study.

## 5. Conclusions

The challenge model with *Eimeria* spp.- and *C. perfringens*-induced dysbiosis negatively affected broiler performance, intestinal permeability, and IL-6 levels. The inclusion of 5% BSF larvae meal in corn–soy diets for broilers mitigated these negative effects but did not lead to significant major improvements. The BSF diet did not adversely impact either the nutrient digestibility or intestinal histomorphology, while it enhanced the cumulative growth performance, increased butyric and acetic acid levels in cecal contents, and elevated lauric and myristic acid concentrations in breast muscle samples. Further research is required to better understand and differentiate the potential functional and antimicrobial properties of BSF larvae meal for poultry.

## Figures and Tables

**Table 1:** Analyzed gross energy and chemical composition of BSF larvae meal.

Item, %	BSF Larvae Meal ^1^
Dry matter	95.0
Gross energy, kcal/kg	5402
Crude protein	52.0
Insect protein corrected for chitin	37.0
Ether extract	16.0
Crude fiber	11.0
Ash	11.0
Calcium	2.0
Total phosphorus	0.68
Available phosphorus	0.65
Total amino acids	
Histidine	1.23
Isoleucine	1.47
Leucine	3.45
Lysine	2.88
Methionine	0.73
Phenylalanine	1.82
Threonine	1.71
Valine	2.27
Tryptophan	0.37
Cysteine	0.35
Tyrosine	2.12
Glycine	1.16

^1^ BSF = Black soldier fly larvae meal (*Hermetia illucens*).

**Table 2:** Ingredient and nutrient composition of experimental diets.

Item	Starter (1 to 14 Days)		Grower (14 to 28 Days)		Finisher (28 to 40 Days)	
	Basal	BSF ^1^	Basal	BSF	Basal	BSF
Ingredient, %						
Corn	58.88	57.81	62.62	60.97	67.30	65.62
Soybean meal	35.79	33.26	32.41	30.45	28.17	26.21
BSF larvae meal	0	5.00	0	5.00	0	5.00
Soybean oil	1.41	0.43	1.50	0.82	1.43	0.73
Dicalcium phosphate	1.17	1.00	1.02	0.85	0.84	0.67
Limestone	1.21	1.01	1.14	0.94	1.05	0.85
Salt	0.52	0.52	0.44	0.44	0.42	0.42
DL-Met, 99%	0.43	0.39	0.31	0.27	0.24	0.20
L-Lys HCl, 78%	0.25	0.08	0.23	0.06	0.22	0.06
L-Thr, 98.5%	0.09	0.03	0.06	0.00	0.05	0.00
L-Val, 98%	0.06	0.00	0.05	0.00	0.04	0.00
Choline chloride, 60%	0.04	0.04	0.06	0.06	0.08	0.08
Vit. and Min. Premix ^2^	0.155	0.155	0.155	0.155	0.155	0.155
Calculated nutrient and energy composition expressed in % or as described						
Metabolizable energy, kcal/kg	2950	2950	3000	3000	3050	3050
Crude protein	22.85	22.85	20.11	20.40	18.49	18.40
Calcium	0.94	0.94	0.88	0.88	0.80	0.80
Available phosphorus	0.45	0.45	0.42	0.42	0.38	0.38
Sodium	0.22	0.22	0.19	0.19	0.18	0.18
Potassium	0.81	0.81	0.79	0.78	0.73	0.72
Chloride	0.41	0.41	0.39	0.39	0.38	0.38
Total choline, mg/kg	1500	1500	1500	1500	1500	1500
Chitin ^3^, g/kg	0	3.50	0	3.50	0	3.50
Dig. Lys	1.22	1.22	1.12	1.12	1.02	1.02
Dig. Met + Cys	0.98	0.98	0.85	0.85	0.75	0.75
Dig. Thr	0.80	0.80	0.74	0.75	0.67	0.69
Dig. Trp	0.25	0.25	0.22	0.22	0.20	0.22
Dig. Arg	1.40	1.40	1.35	1.35	1.10	1.10
Dig. Val	0.94	0.94	0.91	0.91	0.78	0.78
Dig. Ile	0.85	0.85	0.76	0.76	0.69	0.69

^1^ BSF = Black soldier fly larvae meal (*Hermetia illucens*). ^2^ Nutrient composition per kg of feed: vitamin A, 8000 IU; vitamin D3, 2000 IU; vitamin E, 30 IU; vitamin K3, 2 mg; thiamine, 2 mg; riboflavin, 6 mg; pyridoxine, 2.5 mg; cyanocobalamin, 0.012 mg; pantothenic acid, 15 mg; niacin, 35 mg; folic acid, 1 mg; biotin, 0.08 mg; iron, 40 mg; zinc, 80 mg; manganese, 80 mg; copper, 10 mg; iodine, 0.7 mg; and selenium, 0.3 mg. Phytase with 20,000 units of fungal phytase/g. ^3^ Calculated based on ingredient composition.

**Table 3:** The fatty acid profile of the experimental diets.

Item ^1^	Starter (1 to 14 Days)		Grower (14 to 28 Days)		Finisher (28 to 40 Days)	
	Basal	BSF ^2^	Basal	BSF	Basal	BSF
Lauric	0	4.90	0	4.77	0	4.70
Myristic	1.57	2.61	1.30	2.31	1.39	2.01
Palmitic	13.95	15.75	14.07	15.83	13.85	15.61
Palmitoleic	2.19	2.77	3.01	2.63	2.26	2.10
Stearic	2.46	2.54	2.39	2.58	2.54	2.56
Oleic	25.17	23.17	24.44	22.86	25.51	23.73
Rumenic	15.14	16.27	15.35	15.47	16.77	16.79
Linoleic	31.31	23.95	31.31	25.99	29.07	25.71
Alpha-linolenic	3.08	2.51	2.93	2.28	2.86	2.61
Eicosapentaenoic	1.04	1.48	1.09	1.50	1.40	1.49
Unknown MUFA	3.79	3.99	3.80	3.89	4.08	4.66
Total SFA	17.98	25.50	17.76	25.48	18.04	25.09
Total MUFA	31.44	30.29	31.56	29.29	31.85	29.49
Total PUFA	50.58	44.20	50.68	45.23	50.10	45.41

^1^ MUFA = monounsaturated fatty acid; SFA: saturated fatty acid; and PUFA = polyunsaturated fatty acid. ^2^ BSF = black soldier fly larvae meal (*Hermetia illucens*).

**Table 4:** The growth performance of non-challenged or challenged broiler chickens fed either a Basal diet or a diet formulated with 5% black soldier fly (BSF) larvae meal.

Item	Diet		Challenge		SEM	*p*-Value Main Factors		
	Basal	BSF	Non-Challenged	Challenged ^1^		Diet	Challenge	Diet × Challenge
1 to 7 days								
BWG ^2^, g	144	144	159 ^a^	130 ^b^	3.3	0.965	0.001	0.919
FI ^3^, g	191 ^x^	181 ^y^	193 ^a^	177 ^b^	2.7	0.062	0.001	0.602
FCR ^4^	1.323	1.254	1.214 ^b^	1.363 ^a^	0.025	0.115	0.002	0.567
7 to 14 days								
BWG, g	258	262	273 ^a^	247 ^b^	4.0	0.609	0.006	0.093
FI, g	369	359	372 ^a^	356 ^b^	2.9	0.289	0.001	0.341
FCR	1.431 ^a^	1.370 ^b^	1.363 ^b^	1.440 ^a^	0.016	0.027	0.006	0.091
14 to 21 days								
BWG, g	510	526	528 ^a^	507 ^b^	5.2	0.118	0.039	0.462
FI, g	660	655	654	660	7.8	0.676	0.100	0.699
FCR	1.295 ^x^	1.246 ^y^	1.239 ^b^	1.302 ^a^	0.015	0.072	0.026	0.370
21 to 28 days								
BWG, g	712	714	728 ^a^	698 ^b^	7.2	0.882	0.048	0.462
FI, g	1085	1063	1083	1064	12.8	0.354	0.439	0.768
FCR	1.524	1.489	1.488	1.525	0.034	0.369	0.363	0.894
28 to 35 days								
BWG, g	826	841	850	817	10.7	0.481	0.139	0.968
FI, g	1478	1447	1440	1484	14.6	0.241	0.190	0.334
FCR	1.789	1.721	1.694 ^b^	1.816 ^a^	0.017	0.184	0.021	0.462
35 to 40 days								
BWG, g	582	609	606	585	14.0	0.361	0.473	0.376
FI, g	1251	1217	1231	1238	12.0	0.340	0.439	0.768
FCR	2.150 ^x^	1.998 ^y^	2.032	2.116	0.028	0.062	0.297	0.393
1 to 21 days								
BWG, g	913	932	960 ^a^	885 ^b^	8.9	0.103	0.001	0.112
FI, g	1207	1187	1208 ^a^	1149 ^b^	11.2	0.965	0.001	0.919
FCR	1.322 ^a^	1.274 ^b^	1.258 ^b^	1.298 ^a^	0.008	0.002	0.043	0.162
21 to 40 days								
BWG, g	2121	2165	2184 ^a^	2102 ^b^	15.5	0.115	0.005	0.358
FI, g	3794	3709	3735 ^b^	3767 ^a^	29.9	0.609	0.006	0.093
FCR	1.789 ^a^	1.713 ^b^	1.710 ^b^	1.792 ^a^	0.014	0.002	0.001	0.202
1 to 40 days								
BWG, g	3034 ^b^	3097 ^a^	3144 ^a^	2986 ^b^	21.8	0.048	0.001	0.180
FI, g	5000	4896	4980 ^a^	4912 ^b^	32.9	0.118	0.039	0.462
FCR	1.648 ^a^	1.581 ^b^	1.584 ^b^	1.645 ^a^	0.011	0.003	0.009	0.100

^a,b^ Means with different superscripts differ by *p* ≤ 0.05 and were considered significantly different based on the Fisher LSD test. ^x,y^ Means with different superscripts differ by 0.05 < *p* ≤ 0.10 and were considered tendencies. ^1^ Challenge = eye-dropped cocci vaccine at 10× the manufacturer recommendation dose on day 1 and the oral gavage with *Clostridium perfringens* (1 × 10^8^ CFU/bird) at 10 and 14 days of age. ^2^ BWG = body weight gain. ^3^ FI = feed intake. ^4^ FCR = feed conversion ratio.

**Table 5:** The apparent ileal digestibility, intestinal integrity, jejunal histomorphology, and gene expression of non-challenged or challenged broiler chickens at 21 days of age and fed either a Basal diet or a diet formulated with the 5% black soldier fly (BSF) larvae meal.

Item	Diet		Challenge		SEM	*p*-Value Main Factors		
	Basal	BSF	Non-Challenged	Challenged ^1^		Diet	Challenge	Diet Challenge
Intestinal integrity								
FITC-d ^2^, μg/mL	0.114	0.110	0.101 ^b^	0.124 ^a^	0.006	0.852	0.041	0.318
Jejunal gene expression								
Mucin-(MUC2)	1.105	1.262	1.303	1.064	0.08	0.346	0.157	0.879
Interleukin-6 (IL-6)	2.180 ^a^	1.709 ^b^	1.307 ^b^	2.582 ^a^	0.22	0.021	0.002	0.891
Jejunal histomorphology								
Villus height, μm	518 ^y^	544 ^x^	538	523	9.06	0.086	0.378	0.124
Crypt depth, μm	93.1	91.1	93.0	92.5	2.04	0.532	0.903	0.795
Muscular height, μm	142	148	143	148	3.46	0.440	0.507	0.508
Villus–Crypt ratio	5.6 ^y^	6.0 ^x^	5.7	5.9	0.14	0.065	0.577	0.156
Apparent ileal digestibility								
IDE ^3^, kcal/kg	3305	3276	3289	3292	24.8	0.581	0.956	0.957
Dry matter, %	70.1	68.9	70.0	69.0	0.64	0.377	0.465	0.839
Crude protein, %	81.4	80.5	81.0	80.9	0.49	0.423	0.887	0.694
Energy, %	73.1	72.2	72.8	72.4	0.55	0.438	0.744	0.916

^a,b^ Means with different superscripts differ by *p* ≤ 0.05 and were considered significantly different based on the Fisher LSD test. ^x,y^ Means with different superscripts differ by 0.05 < *p* ≤ 0.10 and were considered tendencies. ^1^ Challenge = eye-dropped cocci vaccine at 10× the manufacturer recommendation dose on day 1 and the oral gavage with *Clostridium perfringens* (1 × 10^8^ CFU/bird) at 10 and 14 days of age. ^2^ FITC-d = systemic fluorescein isothiocyanate–dextran. ^3^ IDE = ileal digestible energy.

**Table 6:** The cecal short-chain fatty acids content (mmol/kg) of broiler chickens fed either a Basal diet or a diet formulated with 5% black soldier fly (BSF) larvae meal.

Item	Diet		Challenge		SEM	*p*-Value Main Factors		
	Basal	BSF	Non-Challenged	Challenged ^1^		Diet	Challenge	Diet × Challenge
Short-chain fatty acids, 21 days								
Acetic	42.3 ^b^	54.8 ^a^	52.8	49.30	1.830	0.040	0.324	0.569
Propionic	6.24	6.51	6.33	6.41	0.540	0.810	0.949	0.519
Propanoic	0.79	0.82	0.72 ^b^	0.89 ^a^	0.042	0.789	0.046	0.719
Butyric	9.01 ^b^	12.41 ^a^	11.76	9.70	0.019	0.013	0.115	0.759
Isobutyric	1.29	1.50	1.33	1.45	0.078	0.194	0.453	0.627
Valeric	1.58	1.78	1.75	1.61	0.136	0.497	0.608	0.785
Short-chain fatty acids, 40 days								
Acetic	52.5	57.5	57.20	52.3	2.010	0.205	0.269	0.373
Propionic	13.60	14.2	13.30	14.50	1.260	0.619	0.307	0.308
Propanoic	0.99	1.02	0.92 ^b^	1.09 ^a^	0.053	0.689	0.050	0.225
Butyric	10.30 ^b^	12.90 ^a^	10.70	12.50	0.020	0.030	0.144	0.456
Isobutyric	1.53	1.54	1.43	1.64	0.060	0.932	0.063	0.262
Valeric	1.82	1.83	1.74	1.91	0.132	0.939	0.437	0.978

^a,b^ Means with different superscripts differ by *p* ≤ 0.05 and were considered significantly different based on the Fisher LSD test. ^1^ Challenge = eye-dropped cocci vaccine at 10× the manufacturer recommendation dose on day 1 and oral gavage with *Clostridium perfringens* (1 × 10^8^ CFU/bird) at 10 and 14 days of age.

**Table 7:** The lipid profile of breast samples from broiler chickens at 21 days of age and fed either a Basal diet or a diet formulated with 5% black soldier fly (BSF) larvae meal.

Fatty Acids	Diet		Challenge		SEM	*p*-Value Main Factors		
	Basal	BSF	Non-Challenged	Challenged ^1^		Diet	Challenge	Diet × Challenge
C12:0 Lauric	0.24 ^b^	1.46 ^a^	0.81	0.89	0.138	0.001	0.571	0.081
C14:0 Myristic	0.56 ^b^	1.28 ^a^	0.90	0.95	0.072	0.001	0.474	0.815
C16:0 Palmitic	26.9	27.3	27.0	27.20	0.260	0.505	0.518	0.169
C16:1 Palmitoleic	3.48	3.01	3.22	3.27	0.080	0.164	0.871	0.777
C18:0 Stearic	7.79	8.00	8.00	7.79	0.143	0.479	0.460	0.126
C18:1 Oleic	34.7	34.2	34.0	34.90	0.530	0.623	0.393	0.443
C18:2 Linoleic	23.7	22.7	23.9	22.50	0.440	0.291	0.103	0.922
C20:1 Eicosennoic	0.41	0.44	0.45	0.40	0.027	0.345	0.184	0.464
C18:3 Alpha-linoleic	1.04	0.98	1.07	0.95	0.039	0.392	0.128	0.758
C20:2 Eicosadienoic	0.51	0.53	0.53	0.50	0.037	0.790	0.693	0.382
C20:4 Arachidonic	0.58	0.64	0.59	0.63	0.029	0.301	0.552	0.639
Total fatty acid (%)	1.27	1.30	1.30	1.26	0.051	0.825	0.789	0.788

^a,b^ Means with different superscripts differ by *p* ≤ 0.05 and were considered significantly different based on the Fisher LSD test. ^1^ Challenge = eye-dropped cocci vaccine at 10× the manufacturer recommendation dose on day 1 and oral gavage with *Clostridium perfringens* (1 × 10^8^ CFU/bird) at 10 and 14 days of age.

## Data Availability

The original contributions presented in this study are included in the article. Further inquiries can be directed to the corresponding author.

## Abbreviations

The following abbreviations are used in this manuscript:

AMPs	Antimicrobial properties
BSF	Black soldier fly
BW	Body weight
BWG	Body weight gain
CP	Crude protein
DM	Dry matter
FAMEs	Fatty acid methyl esters
FCR	Feed conversion ratio
FI	Feed intake
FITC-d	Fluorescein isothiocyanate–dextran
IDE	Ileal digestible energy
GC	Gas chromatograph
IFN-	Interferon
IL	Interleukin
MUC	Mucin
MUFA	Medium unsaturated fatty acid
qRT-PCR	Quantitative real-time PCR
SBM	Soybean meal
SCFA	Short-chain fatty acid
